# Factors influencing the acute pentylenetetrazole‐induced seizure paradigm and a literature review

**DOI:** 10.1002/acn3.51375

**Published:** 2021-06-08

**Authors:** Christopher J. Yuskaitis, Leigh‐Ana Rossitto, Karenna J. Groff, Sameer C. Dhamne, Bo Zhang, Lahin K. Lalani, Achint K. Singh, Alexander Rotenberg, Mustafa Sahin

**Affiliations:** ^1^ F.M. Kirby Neurobiology Center Boston Children’s Hospital Harvard Medical School Boston Massachusetts 02115 USA; ^2^ Department of Neurology Boston Children’s Hospital Harvard Medical School Boston Massachusetts 02115 USA; ^3^ Division of Epilepsy and Clinical Neurophysiology and Epilepsy Genetics Program Boston Children’s Hospital Harvard Medical School Boston Massachusetts 02115 USA; ^4^ Biostatistics and Research Design Center Institutional Centers for Clinical and Translational Research Boston Children’s Hospital Harvard Medical School Boston Massachusetts 02115 USA; ^5^ Rosamund Stone Zander Translational Neuroscience Center Boston Children’s Hospital Boston Massachusetts 02115 USA; ^6^ Neuromodulation Program Department of Neurology Boston Children’s Hospital Harvard Medical School Boston Massachusetts 02115 USA

## Abstract

**Objective:**

To confirm the critical factors affecting seizure susceptibility in acute pentylenetetrazole (PTZ) mouse epilepsy models and evaluate the prior literature for these factors.

**Methods:**

Serial cohorts of wild‐type mice administered intraperitoneal (IP)‐PTZ were aggregated and analyzed by multivariate logistic regression for the effect of sex, age, background strain, dose, and physiologic stress (i.e., EEG implantation and/or single‐housing) on seizure response. We assessed the reporting of these factors in a comprehensive literature review over the last 10 years (2010–2020).

**Results:**

We conducted aggregated analysis of pooled data of 307 mice (220 C57BL/6J mice and 87 mixed background mice; 202 males, 105 females) with median age of 10 weeks (range: 6–49 weeks) with acute PTZ injection (dose range 40–65 mg/kg). Significance in multivariate analysis was found between seizures and increased PTZ dose (odds ratio (OR) 1.149, 95% confidence interval (CI) 1.102–1.205), older age (OR 1.1, 95% CI 1.041–1.170), physiologic stress (OR 17.36, 95% CI 7.349–44.48), and mixed background strain (OR 0.4725, 95% CI 0.2315–0.9345). Literature review identified 97 papers using acute PTZ‐seizure models. Age, housing, sex, and background were omitted by 61% (59/97), 51% (49/97), 18% (17/97), and 8% (8/97) papers, respectively. Only 17% of publications specified all four factors (16/97).

**Interpretation:**

Our analysis and literature review demonstrate a critical gap in standardization of acute PTZ‐induced seizure paradigm in mice. We recommend that future studies specify and control for age, background strain, sex, and housing conditions of experimental animals.

## Introduction

Rigor and reproducibility in preclinical epilepsy models is essential for understanding epilepsy and expediting antiseizure drug discovery.^1^, ^2^, ^3^ Mouse models are particularly attractive for epilepsy research, as they can be genetically manipulated to recapitulate the genetic factors associated with many types of epilepsy.^4^, ^5^, ^6^, ^7^, ^8^ In addition to genetic models, mice with single or spontaneous, recurrent seizures can be generated through the use of electrical stimulation or chemo‐convulsants, such as pentylenetetrazole (PTZ), pilocarpine, and kainic acid.^9^, ^10^, ^11^ PTZ specifically has been utilized to model epilepsy since the 1940s and has been a powerful tool for antiseizure drug discovery.^2^, ^12^, ^13^, ^14^ While the mechanism of action is still not fully understood, PTZ is thought to act primarily as a GABA_A_ receptor antagonist to induce seizures, and is employed in both acute and chronic models of epilepsy.^15^, ^16^ In acute paradigms, PTZ injection intraperitoneally (IP), subcutaneously (SubQ), or intravenously (IV) induces a stereotypic seizure response, following the Racine seizure scale and progressing through myoclonic jerking to generalized tonic–clonic seizures (GTCS).^17^ There are multiple endpoints to evaluate seizure susceptibility in a PTZ paradigm, including seizure onset, latency, or duration; dosage threshold; Racine or other behavioral scales; electroencephalogram (EEG) characteristics; and mortality. Chronic epilepsy paradigms administer PTZ repeatedly to induce a kindling effect.^18^, ^19^

Acute PTZ model offers many benefits as a seizure paradigm, including its ease of administration, ability to evoke a full range of seizure types, and utility in drug screening.^20^ However, PTZ has been used inconsistently to induce acute seizures, making it difficult to compare or replicate results across studies and evaluate potential treatments. There are many potential factors that could confound and lead to inconsistencies in prior studies. Strain differences in seizure susceptibility among inbred and outbred strains are well‐documented.^21^, ^22^ Non‐ovariectomized females have a higher seizure threshold to PTZ‐induced seizures,^23^ but this difference may be dose‐dependent.^24^ In rat models, sex and housing did not influence PTZ‐induced seizures.^25^ A recent study has demonstrated single‐housing induces stress in mice and reduces seizure threshold to pilocarpine‐induced seizures.^26^ The effect of housing conditions on PTZ seizure threshold has not been tested in mice. In general, physiologic stress is known to increase seizure susceptibility, but the individual contributions are inconsistently documented in the literature.^27^ EEG implantation increased seizures in the acute setting in a kainic acid seizure paradigm.^28^ Furthermore, it is unclear if prior studies considered these factors in their preclinical research.

To better understand the influences of age, background strain, sex, and physiologic stress on acute PTZ‐induced seizures, we evaluated aggregated seizure response data from several serial cohorts of IP‐injected mice in the Experimental Neurophysiology Core at Boston Children’s Hospital. We then systematically evaluated the literature from the past 10 years to assess if these critical factors were specified and controlled for in acute PTZ paradigm protocols across several studies. Our study and literature review together outline the important factors necessary to specify and control for in acute PTZ‐induced seizure paradigms and serve as a framework for future studies using acute PTZ administration to model epilepsy in mice.

## Methods

### Mice

All mouse procedures were performed in accordance with the Guide for the Humane Use and Care of Laboratory Animals, and the studies included were approved by the Animal Care and Use Committee of Boston Children’s Hospital. All mice were housed in a 12‐h light–dark cycle, climate‐ and humidity‐controlled room, with access to food and water ad libitum.

Littermates and sexes were divided evenly across experimental groups. Cohorts of wild‐type control mice were included in this analysis, some of which have previously been published.^29^, ^30^ Wild‐type mice on both pure C57BL/6J and mixed (129S4/SvJae, C57BL/6J, and CBA) backgrounds were used.^31^ Ages of mice were grouped as follows, in accordance with widely accepted guidelines: pup (P0 to P21), juvenile (3–6 weeks), young adult (6–12 weeks), mature adult (13 weeks to 6 months), middle‐aged adult (10–14 months), and old‐aged adult (over 18 months).^32^ Mice were either socially housed (single‐sex group‐housing for entire life, 2–5 mice per cage) or singly housed (isolated for ≥1 week, as required in EEG‐monitoring or behavioral experiments).

### Acute PTZ seizure paradigm

PTZ (Sigma‐Aldrich, #P6500) was dissolved fresh before each experiment in 0.9% saline at 10 mg/mL. All mice were injected during the light phase of the light–dark cycle. Mice were weighed prior to dosing and injected IP (volume PTZ (mL) = weight (kg) × dose (mg/kg)/10 mg/mL). Post‐injection, mice were placed in individual transparent cages and monitored for seizure over a 10‐min period. After a 10‐min observation period, mice were killed. Our primary endpoint was GTCS, as behaviorally indicated by loss of balance and continuous, uncontrollable jerking (Racine score of 5). A GTCS ended when the animal regained balance or righted itself and ceased jerking (post‐ictal phase) or died. Start and finish time of GTCS and time of death were recorded.

We analyzed aggregated data from 22 both published and unpublished cohorts from the Experimental Neurophysiology Core at Boston Children’s Hospital over a 7‐year period (2013–2020) using the same standardized method for PTZ‐induced seizures. Because these cohorts were collected and measured in comparable ways and between‐cohort heterogeneity is negligible, aggregated analysis of the pooled data was performed.^33^ The benefit of utilizing the larger pooled data set was to critically assess potential factors that influence seizure susceptibility with increased power. We included studies that injected single doses of IP PTZ following the previously outlined protocol, using GTCS as the primary endpoint. We evaluated the contributions of sex, age (young to middle‐aged adults, range: 6–49 weeks), background strain (C57BL/6J v. mixed, as previously described), dose (40–65 mg/kg), and physiologic stress (housing: social v. single; EEG surgery: implanted v. not) to seizure response in an acute PTZ model.

Statistical analyses were performed using GraphPad Prism 8 and RStudio software. All factors in analysis were compared to seizure response. Univariate comparisons were performed by Pearson’s chi‐squared tests for categorical data (sex, stress, and background), Mann‐Whitney U tests for continuous, non‐parametric data (age and dose) and Spearman correlation. Fisher’s exact test and chi‐square test of trend were performed on subgroup analysis. Multivariate comparisons were performed by multivariate logistic regression analysis. A *p*‐value of less than 0.05 was considered statistically significant.

### Literature review

To identify studies that employed acute PTZ as a seizure paradigm, we performed a PubMed search for the 10 years (January 2010–May 2020) using the following search criteria: (pentylenetetrazole[Title/Abstract] OR PTZ[Title/Abstract]) AND mouse[Title/Abstract] AND (seizure[Title/Abstract] OR epilepsy [Title/Abstract]) NOT kindling[Title/Abstract]. Papers were excluded post hoc if they were review articles, kindling models (repetitive injections of PTZ) otherwise not omitted, PTZ paradigms in zebrafish or rat models only, and/or evaluations of PTZ as a treatment only. Our search yielded 130 results, and 97 papers were included in our analysis.

Per reviewed manuscript, we recorded the age, background strain, sex, and housing of experimental mice, and route of PTZ administration, dose, and primary endpoint(s). We systematically evaluated if each of these critical factors were included in the study cohort. A factor was considered unspecified (uns.) if there was no mention of it or there was insufficient detail to replicate the study. Primary endpoints were grouped into categories for simplicity: (1) binomial seizure endpoint (GTCS, other seizure, death), (2) seizure feature (e.g., latency, duration), (3) behavioral scoring, (4) EEG characterization, (5) seizure threshold calculation, (6) other, or (7) undefined/unclear. Ages were grouped as previously explained.

## Results

### Factors influencing PTZ‐induced seizures in mice

We evaluated the contributions of age (young to middle‐aged adults, 6–49 weeks), background strain (C57BL/6J v. mixed, as previously described), sex, physiologic stress (housing: social vs. single; EEG surgery: implanted vs. not), and dose (40–65 mg/kg) to seizure response (Racine scale 5, GTCS) in an acute IP PTZ model. In total, our aggregated analysis of pooled data includes 307 mice from 22 cohorts (median age: 10 weeks, range: 6–49 weeks), 220 C57BL/6J background, 87 mixed background; 202 males, 105 females; 255 unstressed (no surgery, group‐housed), 52 stressed (surgery and/or single‐housed); 160 given 40 mg/kg PTZ, 28 given 48–50 mg/kg PTZ, 63 given 55–57 mg/kg, and 56 given 65 mg/kg (Table S1).

Results of our aggregated analysis are summarized in Tables 1 and 2. In response to IP PTZ injection, stratified by age, 34% of young adult mice seized (76/221), 14% of mature adult mice seized (10/73), and 69% of middle‐aged adult mice seized (9/13). Stratified by background strain, 30% of C57BL/6J mice seized (65/220) and 34% of mixed background mice seized (30/87). Stratified by sex, 30% of male mice seized (61/202) and 32% of female mice seized (34/105). Stratified by physiologic stress, 54% of stressed mice seized (28/52) while only 26% of unstressed mice seized (58/238). Lastly, stratified by dose, 19% of mice administered 40 mg/kg seized (30/160), 54% of mice administered 48–50 mg/kg seized (15/28), 35% of mice administered 55–57 mg/kg seized (22/63), and 50% of mice administered 65 mg/kg seized (28/56). Following this dose–response curve, CD50 for C57BL/6J mice was 65 mg/kg. In our univariate analysis (Table 1), significant correlations were found between seizures and PTZ dose (*p* < 0.0001), age (*p* < 0.027), and physiologic stress (*p* < 0.0001), but not background strain (*p* = 0.4) or sex (*p* = 0.69).

**Table 1:** Univariate analysis of factors affecting acute PTZ‐induced seizures in mice.

	GTCS	No GTCS	*R*	95% Confidence Interval	*p*‐value
Age					
Young adult	34.39% (76/221)	65.61% (145/221)	‐0.1261	‐0.2379 to ‐0.01104	0.027
Mature adult	13.70% (10/73)	86.30% (63/73)			
Middle‐aged adult	69.23% (9/13)	30.77% (4/13)			
Background Strain					
C57BL/6J	29.55% (65/220)	70.45% (155/220)	0.04813	‐0.06746 to 0.1625	0.4
Mixed	34.48% (30/87)	65.52% (57/87)			
Sex					
Male	30.20% (61/202)	69.80% (141/202)	0.02240	‐0.09306 to 0.1373	0.7
Female	32.38% (34/105)	67.62% (71/105)			
Physiologic stress					
Not present	26.27% (67/255)	73.73% (188/255)	0.2237	0.1114 to 0.3304	<0.0001
Present	53.85% (28/52)	46.15% (24/52)			
Dose					
40 mg/kg	18.75% (30/160)	81.25% (130/160)	0.2687	0.1584 to 0.3724	<0.0001
48–50 mg/kg	53.57% (15/28)	46.43% (13/28)			
55–57 mg/kg	34.92% (22/63)	65.08% (41/63)			
65 mg/kg	50.00% (28/56)	50.00% (28/56)			

**Table 2:** Multivariate analysis of factors affecting acute PTZ‐induced seizures in mice.

Dosage	Factor	Odds ratio	95% Confidence interval	*p*‐value
All	Dose	1.149	1.102–1.205	<0.0001
	Gender	0.9512	0.5212–1.722	0.8692
	Age	1.1	1.041–1.170	0.0014
	Physiologic stress	17.36	7.349–44.58	<0.0001
	Background strain	0.4725	0.2315–0.9345	0.0348
40 mg/kg	Gender	0.585	0.1293–2.504	0.4745
	Age	1.169	1.053–1.308	0.0038
	Physiologic stress	60.72	15.94–324.0	<0.0001
	Background strain	0.2363	0.0534–0.8883	0.0409

Multivariate logistic regression model evaluating the associate between the factors and the likelihood of PTZ‐induced seizures. Total cohort (*n* = 307) followed by sub‐analysis of only those mice (*n* = 160) treated with 40 mg/kg PTZ. Odds ratio with associated confidence intervals and *p*‐values are provided.

In multivariate analysis, the main drivers of seizure susceptibility were dose (*p* < 0.0001, OR 1.149, 95% CI 1.102–1.205), older age (*p* = 0.0014, OR 1.1, 95% CI 1.041–1.170), physiologic stress (*p* < 0.0001, OR 17.36, 95% CI 7.349–44.48), and mixed background (*p* = 0.0348, OR 0.4725, 95% CI 0.2315–0.9345) (Table 2). Significant effects of age, stress, and background on seizure susceptibility were maintained in 160 mice when controlling for PTZ dose (40 mg/kg) (Table 2).

**FIGURE 1:**
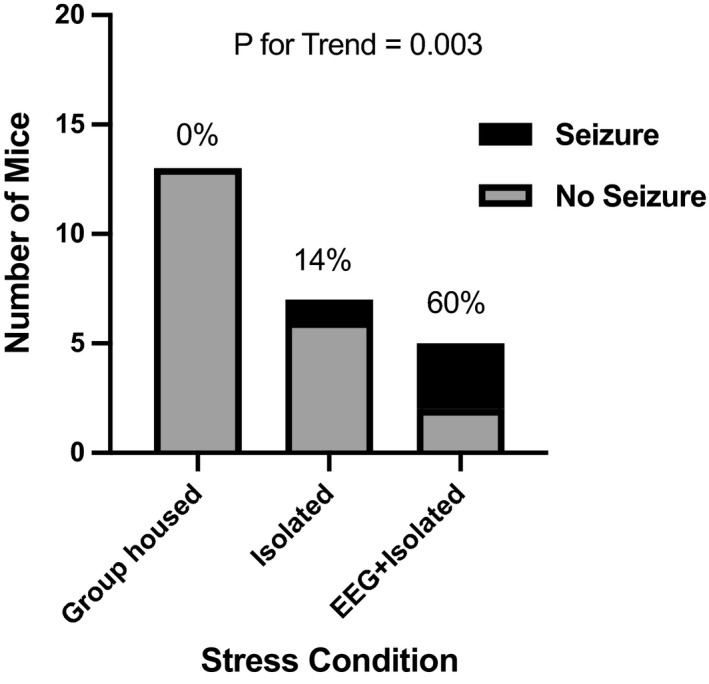
PTZ‐induced seizures are increased with physiologic stressors. Percentage of seizures per group is presented above the bars. **p = 0.003, chi‐square test for trend across the three groups. Mice in the EEG + Isolated cohort have significantly more seizures than the group‐housed cohort as compared by Fisher exact *t*‐test *p* = 0.012.

We performed subgroup analysis for sex and stress. To evaluate sex differences, we analyzed a subgroup of mice where the age, background, stress, and dose were identical and fixed. No significant difference in seizure susceptibility in male and female mice was found within either Cohort 16 (29% (7/24) males and 31% (8/26) females with seizures; *p* > 0.99) or Cohort 19 (50% (12/24) males and 38% (8/21) females with seizures; *p* > 0.57) (Table S1). To evaluate the impact of specific physiologic stressors on increases seizure susceptibility, we analyzed the cohorts with identical age, background, and PTZ‐dose (Cohorts 9, 10, 22, Table S1). We identified a significant correlation between PTZ‐induced seizures and increased stressed conditions from group‐housed mice (0%, 0/13) to isolated mice (14%, 1/7) to isolated mice with EEG implantation (60%, 3/5) (Fig. 1; Chi‐square for trend *p* = 0.003). Collectively, our data confirm age, stress, and background strain are important factors influencing PTZ‐induced seizure susceptibility in wild‐type mice.

### Literature review of PTZ‐induced seizures in mice

**FIGURE 2:**
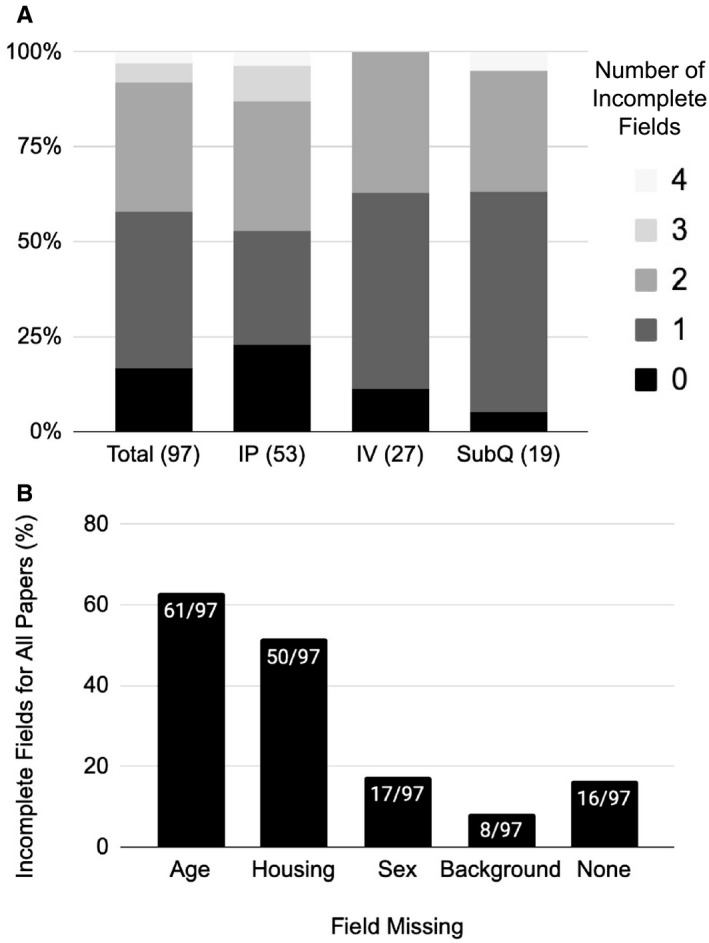
Critical factors incompletely specified in PTZ‐related epilepsy papers. (A) Stacked column chart showing percentage of literature review papers that omitted 0, 1, 2, 3, or 4 of the critical factors identified (age, physiologic stressor, sex, and background). Findings are stratified based upon the route of PTZ administration (IP, IV, or SubQ). (B) Bar chart demonstrating the percentage of literature review papers that incompletely specified each critical factor.

Our data highlight the importance of accounting for baseline characteristics in PTZ‐induced seizure mouse models of epilepsy. We undertook a comprehensive literature review to evaluate prior studies using acute PTZ‐induced seizures in mouse models. We identified 97 publications over the past 10 years (2010–2020). Our findings are summarized in Table 3 (full review in Table S2) and Figure 2. The most common route of acute PTZ administration was IP (55%, 53/97), followed by IV (28%, 27/97), then SubQ (20%, 19/97). Two studies used both IP and IV methods. The most common background strain was C57BL/6 (all sub‐strains: 40%, 39/97), with BL/6J the most common specified substrain of BL/6 (46%, 18/39) and 41% using an unspecified substrain of C57BL/6 (16/39). Other common background strains were CD‐1/Swiss Albino (23%, 22/97) and NMRI (11%, 11/97). A majority of studies used adult mice (54%, 52/97), 22% with specified ages in the young adult range (21/97), 14% with specified ages in the mature adult range (14/97), 2% with specified ages in the middle‐aged adult range (2/97), 18% with adults of unspecified ages (17/97). Only 3% of studies used juvenile mice (3/97). A large majority of studies used only male mice (72%, 70/97), whereas 5% used only females (5/97) and 5% used both males and females (5/97). Group‐housing conditions were specified by 37% (36/97) and single‐housing by 12% (12/97) of studies.

**Table 3:** Literature review of factors included in papers using PTZ‐induced acute seizure models in mice.

Age^1^	Background	Sex	Housing	Dose	GTCS	Other Sz^2^	Death	Sz Features^3^	Sz Scoring^4^	EEG	Threshold^5^	Other	Unclear
Pup	C57BL/6J	Uns.	Group	40 & 90 mg/kg	+	−	−	−	−	−	−	−	−
	CD‐1	Uns.	Uns.	50 & 100 mg/kg	−	−	−	−	+	−	−	+	−
Juvenile	Albino	M	Uns.	60 mg/kg	−	−	−	+	+	−	−	−	−
Young, uns.	CD‐1	M	Group	60 mg/kg	+	−	+	−	+	−	−	−	−
Young adult	BALB/c	M	Uns.	61.96 mg/kg	−	−	−	−	−	−	+	−	−
	C57BL/6	F	Group	50 & 80 mg/kg	−	−	−	+	+	−	−	−	−
		M	Group	45 mg/kg	−	−	−	+	−	+	−	−	−
				50 mg/kg	−	−	−	−	+	−	−	−	−
			Uns.	50 mg/kg	−	+	+	−	−	+	−	−	−
		Uns.	Uns.	60 mg/kg	−	−	−	−	+	−	−	−	−
	C57BL/6J	M	Single	80 mg/kg	+	+	−	−	+	−	−	−	−
			Uns.	45 mg/kg	−	−	−	−	+	+	−	−	−
				60 mg/kg	−	−	−	−	+	−	−	−	−
		Uns.	Uns.	75 mg/kg	−	−	+	−	−	−	−	−	−
	C57BL/6N	M + F	Uns.	60 mg/kg	−	−	+	+	+	−	−	−	−
	KunMing	M	Group	60 mg/kg	−	−	−	+	+	+	−	−	−
	Mixed	M	Single	40–70 mg/kg	+	+	−	+	+	+	−	−	−
	Uns.	M	Uns.	85 mg/kg	−	−	−	−	+	−	−	−	−
Mature adult	C57BL/6SJL	F	Group	40 mg/kg	−	−	−	−	+	−	−	−	−
	C57BL/6	M	Single	50 mg/kg	−	+	+	−	+	−	−	−	−
	C57BL/6J	M	Single	75 mg/kg	−	−	−	−	−	−	−	−	+
		M + F	Group	60 mg/kg	−	−	−	+	−	−	−	−	−
			Uns.	50 mg/kg	−	+	+	+	−	+	−	−	−
	Uns.	M	Uns.	50 mg/kg	+	−	−	+	−	−	−	−	−
				55 mg/kg	+	−	+	+	−	−	−	−	−
Adult, uns.	Albino	M	Uns.	105 mg/kg	−	+	−	−	−	−	−	−	−
	C57BL/6	M	Uns.	30, 55, & 75 mg/kg	−	−	−	+	−	−	−	−	−
	C57BL/6S	M	Single	50 mg/kg	−	−	−	+	+	+	−	−	−
	CD‐1	F	Uns.	60 mg/kg	−	−	−	−	−	−	−	+	−
		M	Uns.	70 mg/kg	−	+	−	−	−	−	−	−	−
	Mixed	M + F	Uns.	50 mg/kg	−	+	−	+	−	−	−	−	−
	Uns.	Uns.	Uns.	55 & 80 mg/kg	−	−	−	−	+	−	−	−	−
	NMRI	M	Group	85 mg/kg	−	−	−	+	−	−	−	−	−
			Uns.	60 mg/kg	+	−	−	−	−	−	−	−	−
Multiple ages	C57BL/6J	M	Group	10–80 mg/kg	−	−	−	−	+	−	−	−	−
					−	−	−	−	+	+	−	−	−
Uns.	C57BL/6	M	Single	30 mg/kg	−	−	−	+	+	−	−	−	−
		Uns.	Uns.	50 mg/kg	−	−	−	−	+	−	−	−	−
	C57BL/6J	Uns.	Single	40 mg/kg	−	+	−	−	−	−	−	−	−
				75 mg/kg	+	−	+	−	+	−	−	−	−
			Group	40 mg/kg	−	−	−	+	−	+	+	−	−
			Uns.	75 mg/kg	−	−	+	−	−	−	−	−	−
	CD‐1	F	Single	85 mg/kg	−	+	−	−	+	−	−	−	−
		M	Group	65 mg/kg	+	−	+	−	−	−	−	−	−
			Uns.	60 mg/kg	+	−	+	−	+	−	+	−	−
				75 mg/kg	+	−	+	−	−	−	−	−	−
				85 mg/kg	+	−	+	+	−	−	−	−	−
	ddY	M	Group	100 mg/kg	−	+	−	−	−	−	−	−	−
	Mixed	Uns.	Uns.	40 mg/kg	−	−	−	+	−	+	−	−	−
	Uns.	Uns.	Single	40 mg/kg	−	−	−	−	−	−	−	+	+
				50 mg/kg	+	−	−	+	−	−	−	−	−
			Uns.	30 mg/kg	−	−	−	−	+	−	−	−	−
	NMRI	M	Group	89 mg/kg	−	+	−	−	−	−	−	−	−

Abbreviations: EEG, Electroencephalogram; Sz, Seizure; Uns., Unspecified.

^1^ Pup: 0–3 weeks, Juvenile: 3–6 weeks, Young adult: 6–12 weeks, Mature adult: 3–6 months.

^2^ For example tonic seizures and myoclonic jerking.

^3^ That is latency, duration, or frequency.

^4^ For example Racine or other behavioral scoring.

^5^ That is a mathematical calculation of seizure threshold.

Primary seizure endpoints were utilized in most of the prior literature. Binomial endpoints (e.g., seizure vs. no seizure) were used in 41% (40/97) of studies, with 20% using GTCS (19/97), 22% using a different seizure type (21/97), and 17% using death (16/97). Other endpoints reported were seizure features (35%, 34/97), seizure rating scale (30%, 29/97), EEG features (10%, 10/97), and seizure threshold (32%, 31/97). Seizure threshold was employed by almost all IV papers (93%, 25/27). In the studies utilizing the IP route, the most common primary endpoints were either binomial seizure/no seizure (27%, 26/97) or seizure scoring scales (26%, 25/97). Forty‐nine percent (47/97) of papers included more than one primary seizure endpoint.

We assessed the included publications for specification of age, background, sex, and housing conditions of the experimental mice (Fig. 2). Only 17% (16/97) of papers in the prior literature specified all four of these critical factors. One, two, or three factors were omitted by 41% (40/97), 34% (33/97), and 8% (8/97) papers, respectively. Age was the most commonly omitted factor, with 60% (59/97) of papers not specifying an age range in their methods. Housing, sex, or background were omitted by 51% (49/97), 18% (17/97), and 8% (8/97) papers, respectively. Within the IP PTZ papers specifically, similar trends were noted with only 23% (12/53) papers including all four critical factors. Again, age was the most commonly omitted factor in the methods, with (51%, 27/53) of papers not specifying an age range. Housing, sex, or background were omitted by (51%, 27/53), 26% (14/57), 13% (7/53) of papers, respectively. Taken together, the majority of published studies failed to explicitly state critical factors that influence PTZ‐induced seizure susceptibility in acute mouse models of epilepsy.

## Discussion

The purpose of this study was two‐fold: (1) to evaluate the influences of age, background strain, sex, and physiologic stress on seizure susceptibility in an acute PTZ epilepsy mouse model through aggregated analysis, and (2) to critically examine the way the field has accounted for these factors over the last 10 years. In our study, we identified dose, age, and stress as critical factors with significant influences on seizure susceptibility in an acute PTZ paradigm. In our literature review, we found that acute PTZ models are inconsistent across studies. Due to varying endpoints and most studies not explicitly stating confounding factors, it is difficult to compare or replicate results and evaluate potential treatments. Together, our aggregated analysis and comprehensive literature review demonstrate a critical gap in standardizing the acute PTZ‐induced seizure paradigm to study epilepsy. It is likely our findings on age, background strain, sex, physiologic stress, and dose will generalize to other ages, strains, and PTZ routes of administration (IV and SubQ) not employed in our study, but more research is necessary to evaluate the role of factors we do not use in our labs.

The American Epilepsy Society (AES) and International League Against Epilepsy (ILAE) Translational Task Force have begun a systematic review of animal research in epilepsy.^34^ This systematic review excludes acute seizure models. Therefore, we evaluated confounds of the acute PTZ‐induced seizure method and evaluated the rigor of prior studies. Our initial goal was to perform a traditional meta‐analysis of the prior literature to evaluate the confounding effects of critical factors (age, sex, physiologic stress, and background strain). However, in our initial literature review, we found the majority of papers omitting at least one of the four critical factors. We evaluated the importance of these factors in our cross‐sectional analysis of the cohorts from our institution and confirmed the importance of these factors. The greatest strength of our study was an increased sample size and the standardization of the method used across our laboratories.

The most striking finding of our study is the influence of physiologic stress on seizure susceptibility, which we represented through single‐housing and EEG implantation surgeries. Mice exposed to physiologic stress before a PTZ seizure challenge seized twice as often as control mice. Although our study was underpowered to directly address the effect of social isolation on seizures, our findings are consistent with other acute seizure paradigms in rodent models. Social isolation increased seizure susceptibility after pilocarpine‐induced seizures.^26^ In a rat study, housing influenced seizure susceptibility to bicuculline.^25^ Future studies should explicitly evaluate the effect of social isolation in PTZ‐induced seizures in multiple background strains of wild‐type mice. In prior literature, housing conditions were not explicitly detailed in over half of the acute PTZ studies from the last decade. It is therefore crucial for the interpretation and replication of results that housing conditions are specified as either group (social) or single (isolated) when using the acute PTZ paradigm.

A limitation of our study is that most of the animals were socially isolated before PTZ administration due to EEG electrode implantation surgery that involved acute exposure to anesthesia. The influence of EEG electrode implantation on seizure susceptibility in mice is poorly understood. Per lab protocol, implanted mice were post‐operatively administered analgesics and allowed to heal for at least a week before any further experiments could be conducted^29^, ^30^, ^31^. One study employing a kainic acid seizure paradigm found that EEG implantation increased seizure endpoints (specifically mortality) regardless of experimental groups.^28^ This paper has two key differences from our study, however: (1) they use juvenile mice, and (2) their postoperative recovery period is much shorter (3 days). They suggest that increased seizure susceptibility is due to inflammation, which may or may not resolve at a later time point. Our data point toward a trend to increased seizure susceptibility in mice that are socially isolated and/or implanted with EEG electrodes. While we cannot exclude the fact that other stressors may also contribute to increased seizure susceptibility, our evidence indicates a graded increase seizure susceptibility to social isolation and EEG implantation in a PTZ‐seizure paradigm. Consistent with prior studies, we found stress increases seizure susceptibility.^27^ Ultimately, more research is needed to understand the mechanisms by which EEG implantation and social isolation alter seizure susceptibility in mice.

In addition, we identified age as a significant factor in seizure susceptibility in both our univariate and multivariate analyses. Our findings on age are limited in scope, as 72% of our mice fall in the young adult category, and our age range is restricted to 6–49 weeks. Even with this restricted age range, we still found a significant influence of age on seizure susceptibility. At the same time, age was the most commonly omitted critical factor in the literature, with many papers omitting age all together and others only specifying the mice as “adult.” Our results provide evidence that even small differences in age result in variability in seizure susceptibility. All studies should report the age range of mice used and restrict the range of ages as much as possible. The underlying mechanisms of seizure susceptibility across ages are not well‐defined, and further research is needed in this area.

We did not identify sex as a significant contributor to seizure susceptibility in wild‐type mice in an acute PTZ paradigm. Most studies did specify the sex of their experimental mice in their methods, and of these studies, a majority used only males. While we did not find sex differences in our study, it would be hasty to generalize these findings to all possible experimental designs, and, therefore, the sex breakdown of an experimental cohort is essential in any acute PTZ methods section. Sex differences are well‐documented in aging and maturation of mice.^35^ We did not track estrous cycles in this study, but previous studies suggest an influence of estrogen and estrous cycles on seizure susceptibility in rodent models.^36^ Some papers in our literature review used this as justification for including only male mice in their studies. It is possible that any effects of estrous cycling were negated by our large sample size. It is also possible that there is no overt sex difference in untreated, wild‐type mice with PTZ‐induced seizures with a large sample size, but sex differences may be evident after treatments or genetic mutant models. Collectively, we are hesitant to conclude whether or not sex plays a role in PTZ‐induced seizure susceptibility, as more nuanced effects may have been averaged out in our analysis.

Other studies have shown that background strain influences seizure presentation and susceptibility.^21^, ^22^ Our results are limited in their scope as we only evaluated C57BL/6J and a single mixed background strain. We found a significant difference in seizure susceptibility in the pure C57BL/6J and mixed background mice in our multivariate analysis. In our literature review, CD‐1/Swiss Albino mice appear to have an increased seizure susceptibility with seizure rates of 100% in all studies reviewed. Additionally, 16 papers identified their mice as C57BL/6, which no longer indicates an actual strain. C57BL/6 requires a laboratory code at the end which indicates the substrain (e.g., J is The Jackson Laboratory and N is the NIH).^37^, ^38^ Based on our findings, we recommend that all studies employing PTZ as an acute paradigm specify fully the background strain of their experimental mice.

## Conclusion

The cohorts included in our study used the most common acute PTZ method of administration (IP), primary endpoint (binomial seizure endpoint), and background strain (C57BL/6J), making our findings useful for a majority of the field. We show that dose, age, background strain, and physiologic stress all influence seizure susceptibility. Physiologic stressors of surgery and housing conditions influence PTZ‐induced seizures, but the mechanisms of these effects remain to be determined. As proposed by the AES‐ILAE report on common data elements, our data highlight the importance of including age, background strain, sex, and housing to improve the rigor and reproducibility of preclinical models of epilepsy.^39^

## Conflicts of Interest

M.S. reports grant support from Novartis, Roche, Pfizer, Biogen, Ipsen, LAM Therapeutics, Astellas, Bridgebio and Quadrant Biosciences. He has served on Scientific Advisory Boards for Sage, Roche, Celgene, Aeovian, Regenxbio and Takeda. None of the other authors has any conflict of interest to disclose.

## Ethical Publication Statement

We confirm that we have read the Journal’s position on issues involved in ethical publication and affirm that this report is consistent with those guidelines.

## Supporting information


**Table S1**. Cohorts of mice used for multivariate analysis of variables related to PTZ‐induced seizures.
**Table S2**. Summary of literature review for intravenous and subcutaneous routes of PTZ‐induced seizures in mice.

**Table S3**. Full literature review separated by route of PTZ administration.
