# Supplementary material for: Factors influencing the acute pentylenetetrazole‐induced seizure paradigm and a literature review
Source: Ann Clin Transl Neurol. 2021 Jun 8;8(7):1388–97. doi: 10.1002/acn3.51375 (PMC8283168; doi:10.1002/acn3.51375)
Supplement: Supplementary file 1 — Table S1. Cohorts of mice used for multivariate analysis of variables related to PTZ‐induced seizures. Table S2. Summary of literature review for intravenous and subcutaneous routes of PTZ‐induced seizures in mice. [file ACN3-8-1388-s002.docx]

**Supporting Information**

Table S1: Cohorts of mice used for multivariate analysis of variables related to PTZ-induced seizures.

| **Cohort** | **Dose (mg/kg)** | **Age (weeks)** | **Background** | **Males (n)** | **Females (n)** | **Physiologic Stress** | **Cohort Seizures (%)** |
| --- | --- | --- | --- | --- | --- | --- | --- |
| Cohort 1 | 40 | 8-10 | C57BL/6J | 5 | 0 | Single+EEG | 100% (5/5) |
| Cohort 2 | 40 | 8-10 | C57BL/6J | 7 | 0 | Single+EEG | 71% (5/7) |
| Cohort 3 | 40 | 8-10 | C57BL/6J | 7 | 0 | Single+EEG | 100% (7/7) |
| Cohort 4 (1) | 40 | 8-10 | C57BL/6J | 7 | 0 | Single+EEG | 0% (0/7) |
| Cohort 5 | 40 | 32 | C57BL/6J | 4 | 3 | Group | 86% (6/7) |
| Cohort 6 | 50 | 10-12 | C57BL/6J | 11 | 0 | Group | 100% (11/11) |
| Cohort 7 | 50 | 10-12 | C57BL/6J | 8 | 0 | Single+EEG | 88% (7/8) |
| Cohort 8 (2) | 40 | 10 | C57BL/6J | 32 | 0 | Group | 9% (3/32) |
| Cohort 9 | 40 | 10-15 | Mixed | 1 | 12 | Group | 0% (0/13) |
| Cohort 10 | 40 | 10-15 | Mixed | 7 | 0 | Single Only | 14% (1/7) |
| Cohort 11 | 40 | 14-15 | C57BL/6J | 0 | 6 | Single+EEG | 33% (2/6) |
| Cohort 12 (2) | 40 | 16 | C57BL/6J | 48 | 0 | Group | 0% (0/48) |
| Cohort 13 | 40 | 6-12 | C57BL/6J | 3 | 3 | Group | 0% (0/6) |
| Cohort 14 | 40 | 6-12 | Mixed | 1 | 9 | Group | 0% (0/10) |
| Cohort 15 | 48 | 6-12 | C57BL/6J | 4 | 5 | Group | 11% (1/9) |
| Cohort 16 | 57 | 6-12 | C57BL/6J | 24 | 26 | Group | 32% (16/50) |
| Cohort 17 | 57 | 15-16 | C57BL/6J | 5 | 5 | Group | 50% (5/10) |
| Cohort 18 | 65 | 6-12 | C57BL/6J | 3 | 4 | Group | 57% (4/7) |
| Cohort 19 | 65 | 6-12 | Mixed | 24 | 21 | Group | 44% (20/45) |
| Cohort 20 | 55 | 15-31 | Mixed | 2 | 1 | Group | 67% (2/3) |
| Cohort 21 | 65 | 33-49 | Mixed | 2 | 2 | Group | 100% (4/4) |
| Cohort 22 | 40 | 10-16 | Mixed | 2 | 3 | Single+EEG | 60% (3/5) |
|  |  |  |  |  |  |  |  |
| 1 Published: Dhamne SC, Silverman JL, Super CE, Lammers SHT, Hameed MQ, Modi ME, Copping NA, | | | | | | |  |
| Pride MC, Smith DG, Rotenberg A, Crawley JN, Sahin M. *Replicable in vivo physiological and behavioral* | | | | | | |  |
| *phenotypes of the Shank3B null mutant mouse model.* Molecular Autism. 2017 Jun 15; 8:26 | | | | | | |  |
| 2 Published: MacMullin P, Hodgson N, Damar U, Lee HHC, Hameed MQ, Dhamne SC, Hyde D, Conley G, | | | | | | |  |
| Morriss N, Qiu J, Mannix R, Hensch TK, Rotenberg A. *Increase in Seizure Susceptibility After Repetitive* | | | | | | |  |
| *Concussion Results from Oxidative Stress, Parvalbumin-Positive Interneuron Dysfunction and Biphasic* | | | | | | |  |
| *Increases in Glutamate/GABA Ratio*. Cerebral Cortex. 2020 Jul 17 | | | | |  |  |  |

Table S2: Summary of literature review for intravenous and subcutaneous routes of PTZ-induced seizures in mice.

| **Route** | **Age^1^** | **Background** | **Sex** | **Housing** | **Dose** | | **GTCS** | **Other Sz ^2^** | **Death** | **Sz Features ^3^** | **Sz Scoring ^4^** | **EEG** | | **Threshold ^5^** | **Other** | **Unclear** |
| --- | --- | --- | --- | --- | --- | --- | --- | --- | --- | --- | --- | --- | --- | --- | --- | --- |
| Intravenous | Young adult | C57BL/6 | M | Single | 7.5 mg/mL at 150 µL/min | | - | - | - | + | - | - | | + | - | - |
|  |  |  |  | Uns. | 7.5 mg/mL at 150 µL/min | | - | - | - | - | - | - | | + | - | - |
|  |  |  |  |  |  | | - | - | - | + | - | - | | + | - | - |
|  |  |  |  |  |  | | - | - | - | + | - | - | | + | - | - |
|  | Mature adult | C57BL/6J | M | Uns. | 1% PTZ at 0.2 mL/min | | - | + | - | - | - | - | | - | - | - |
|  |  | C57BL/6N | M | Group | 10 mg/mL at 100 µL/min | | - | - | - | - | - | - | | + | - | - |
|  |  |  |  |  | 10 mg/mL at uns. rate | | + | - | - | - | - | - | | + | - | - |
|  | Adult, uns. | CD-1 | M | Uns. | 10 mg/mL at 150 µL/min | | - | - | - | - | - | - | | + | - | - |
|  |  |  |  |  |  | | - | - | - | - | - | - | | + | - | - |
|  |  |  |  |  |  | | - | - | - | + | - | - | | + | - | - |
|  |  | CF-1 Albino | M | Uns. | 10 mg/mL at 150 µL/min | | - | - | - | + | - | - | | + | - | - |
|  |  | NMRI | M | Group | 0.5% PTZ at 0.5 mL/min | | - | - | - | - | - | - | | + | - | - |
|  |  |  |  | Uns. | 10 mg/mL at 100 µL/min | | - | - | - | - | - | - | | + | - | - |
|  | Uns. | Albino | M | Uns. | 0.5% PTZ at 1 mL/min | | - | - | - | + | - | - | | + | - | - |
|  |  | C57BL/6 | M | Group | 7.5 mg/mL at 150 µL/min | | - | - | - | + | + | - | | - | - | - |
|  |  | CD-1 | M | Uns. | 1% PTZ at 0.2 mL/min | | - | - | - | - | - | - | | + | - | - |
|  |  |  |  |  |  | | - | - | - | - | - | - | | + | - | - |
|  |  |  |  |  | 10 mg/mL at 0.05 mL/min | | - | - | - | - | - | - | | + | - | - |
|  |  | ICR | M | Group | 10 mg/mL at 0.005 mL/s | | - | - | - | - | - | - | | + | - | - |
|  |  | NMRI | F | Group | 1% PTZ at 0.3 mL/min | | - | + | - | - | - | - | | + | - | - |
|  |  |  | M | Group | 0.5% at 1 mL/min | | - | - | - | - | - | - | | + | - | - |
|  |  |  |  |  |  | | - | - | - | - | - | - | | + | - | - |
|  |  |  |  |  | 0.5% PTZ at 0.5 mL/min | | - | - | - | - | - | - | | + | - | - |
|  |  |  |  |  |  | | - | - | - | - | - | - | | + | - | - |
|  |  |  |  |  | 7.5 mg/mL at 150 µL/min | | - | - | - | - | - | - | | + | - | - |
|  |  |  |  |  |  | | - | - | - | + | - | - | | + | - | - |
|  |  |  |  | Uns. | 0.5% PTZ at 1 mL/min | | - | - | - | - | - | - | | + | - | - |
|  |  |  |  |  |  | |  |  |  |  |  |  | |  |  |  |
| Subcutaneous | Young adult | C57BL/6 | M | Uns. | 90 mg/kg | | + | - | + | - | - | - | | - | + | - |
|  |  | C57BL/6J | M+F | Uns. | 60-90 mg/kg | | + | - |  | + | - | - | | + | - | - |
|  |  |  | Uns. | Uns. | 100 mg/kg | | - | - | - | + | - | - | | - | - | - |
|  | Mature adult | C57BL/6 | M | Group | 60 mg/kg | | - | - | - | - | + | - | | - | - | - |
|  |  | CD-1 | M | Uns. | 40 mg/kg | | - | - | - | - | + | - | | - | - | - |
|  | Adult, uns. | CD-1 | M | Group | 100 mg/kg | | + | - | + | - | - | - | | - | - | - |
|  |  |  |  |  |  | | + | + | - | + | - | - | | - | - | - |
|  |  |  |  |  | 50-100 mg/kg | | + | - | - | - | + | - | | - | - | - |
|  |  | FVB/N | M | Single | 50 mg/kg | | - | - | - | + | - | - | | + | - | - |
|  | Uns. | BALB/e | M | Group | 85 mg/kg | | - | + | + | - | - | - | | - | - | - |
|  |  | C57BL/6J | M | Uns. | 85 mg/kg | | - | - | - | + | - | - | | - | - | - |
|  |  | CD-1 | M | Group | 100 mg/kg | | - | - | - | + | - | - | | - | - | - |
|  |  |  |  |  | 80 mg/kg | | - | + | - | - | - | - | | - | - | - |
|  |  |  |  | Uns. | 50-110 mg/kg | | - | + | - | - | - | - | | + | - | - |
|  |  | CF-1 Albino | M | Uns. | 85 mg/kg | | - | + | - | - | - | - | | - | - | - |
|  |  | ICR | M | Group | 65 mg/kg | | - | - | - | + | - | - | | - | - | - |
|  |  |  |  | Uns. | 90 mg/kg | | - | + | - | - | - | - | | - | - | - |
|  |  | KunMing | Uns. | Group | 85 mg/kg | | - | - | - | - | - | - | | - | - | + |
|  |  | Uns. | Uns. | Uns. | 85 mg/kg | | - | + | - | - | - | - | | - | - | - |
| Abbreviations: Unspecified (Uns.), Seizure (Sz), Electroencephalogram (EEG) | | | | | | | | | | | | |  |  |  |  |
| 1 Pup: 0-3 weeks, Juvenile: 3-6 weeks, Young adult: 6-12 weeks, Mature adult: 3-6 months | | | | | | | | | | | | | | | | |
| 2 e.g. tonic seizures and myoclonic jerking | | | | | |  | | |  |  |  |  |  |  |  |  |
| 3 i.e. latency, duration, or frequency | | | | | |  | | |  |  |  |  |  |  |  |  |
| 4 e.g. Racine or other behavioral scoring | | | | | |  | | |  |  |  |  |  |  |  |  |
| 5 i.e. a mathematical calculation of seizure threshold | | | | | | | | |  |  |  |  |  |  |  |  |

**Supplemental References**

1. Aghamiri H, Shafaroodi H, Asgarpanah J. Anticonvulsant Activity of Essential Oil From Leaves of Zhumeria majdae (Rech.) in Mice: The Role of GABAA Neurotransmission and the Nitric Oxide Pathway. Clinical and Translational Science. 2020; 13(4):785–97.
2. Aguiar CCT, Almeida AB, Araújo PVP, Vasconcelos GS, Chaves EMC, do Vale OC, et al. Anticonvulsant effects of agomelatine in mice. Epilepsy Behav. 2012; 24(3):324–8.
3. Amini-Khoei H, Kordjazy N, Haj-Mirzaian A, Amiri S, Haj-Mirzaian A, Shirzadian A, et al. Anticonvulsant effect of minocycline on pentylenetetrazole-induced seizure in mice: involvement of nitric oxide and N-methyl-d-aspartate receptor. Can J Physiol Pharmacol. 2018; 96(8):742–50.
4. Amini-Khoei H, Rahimi-Balaei M, Amiri S, Haj-Mirzaian A, Hassanipour M, Shirzadian A, et al. Morphine modulates the effects of histamine H1 and H3 receptors on seizure susceptibility in pentylenetetrazole-induced seizure model of mice. European Journal of Pharmacology. 2015; 769:43–7.
5. Andres-Mach M, Zolkowska D, Barcicka-Klosowska B, Haratym-Maj A, Florek-Luszczki M, Luszczki JJ. Effect of ACEA—a selective cannabinoid CB1 receptor agonist on the protective action of different antiepileptic drugs in the mouse pentylenetetrazole-induced seizure model. Progress in Neuro-Psychopharmacology and Biological Psychiatry. 2012; 39(2):301–9.
6. Auer T, Schreppel P, Erker T, Schwarzer C. Functional characterization of novel bumetanide derivatives for epilepsy treatment. Neuropharmacology. 2020; 162:107754.
7. Bezzina C, Verret L, Halley H, Dahan L, Rampon C. Environmental enrichment does not influence hypersynchronous network activity in the Tg2576 mouse model of Alzheimer’s disease. Front Aging Neurosci. 2015; 7:178.
8. Bodda C, Tantra M, Mollajew R, Arunachalam JP, Laccone FA, Can K, et al. Mild Overexpression of Mecp2 in Mice Causes a Higher Susceptibility toward Seizures. The American Journal of Pathology. 2013; 183(1):195–210.
9. Bolkvadze T, Pitkänen A. Development of Post-Traumatic Epilepsy after Controlled Cortical Impact and Lateral Fluid-Percussion-Induced Brain Injury in the Mouse. Journal of Neurotrauma. 2011; 29(5):789–812.
10. Bolkvadze T, Puhakka N, Pitkänen A. Epileptogenesis after traumatic brain injury in Plaur-deficient mice. Epilepsy & Behavior. 2016; 60:187–96.
11. Brault V, Martin B, Costet N, Bizot J-C, Hérault Y. Characterization of PTZ-Induced Seizure Susceptibility in a Down Syndrome Mouse Model That Overexpresses CSTB. PLOS ONE. 2011; 6(11):e27845.
12. Buenafe OE, Orellana-Paucar A, Maes J, Huang H, Ying X, De Borggraeve W, et al. Tanshinone IIA Exhibits Anticonvulsant Activity in Zebrafish and Mouse Seizure Models. ACS Chem Neurosci. 2013; 4(11):1479–87.
13. Cao L, Tian Y, Jiang Y, Zhang G-J, Lei H, Di Z-L. Down-Regulation of Homer1b/c Protects Against Chemically Induced Seizures Through Inhibition of mTOR Signaling. Cell Physiol Biochem. 2015; 35(4):1633–42.
14. Carrasco‐Pozo C, Tan KN, Borges K. Sulforaphane is anticonvulsant and improves mitochondrial function. Journal of Neurochemistry. 2015; 135(5):932–42.
15. Chen CR, Tan R, Qu WM, Wu Z, Wang Y, Urade Y, et al. Magnolol, a major bioactive constituent of the bark of Magnolia officinalis, exerts antiepileptic effects via the GABA/benzodiazepine receptor complex in mice. British Journal of Pharmacology. 2011; 164(5):1534–46.
16. Cheng Y-Y, Chou Y-T, Lai F-J, Jan M-S, Chang T-H, Jou I-M, et al. Wwox deficiency leads to neurodevelopmental and degenerative neuropathies and glycogen synthase kinase 3β-mediated epileptic seizure activity in mice. Acta Neuropathologica Communications. 2020; 8(1):6.
17. Cinar N, Sahin S, Erdinc OO. What is the impact of electromagnetic waves on epileptic seizures? Med Sci Monit Basic Res. 2013; 19:141–5.
18. 18. Citraro R, Navarra M, Leo A, Donato Di Paola E, Santangelo E, Lippiello P, et al. The Anticonvulsant Activity of a Flavonoid-Rich Extract from Orange Juice Involves both NMDA and GABA-Benzodiazepine Receptor Complexes. Molecules. 2016; 21(9):1261.
19. Copmans D, Orellana-Paucar AM, Steurs G, Zhang Y, Ny A, Foubert K, et al. Methylated flavonoids as anti-seizure agents: Naringenin 4′,7-dimethyl ether attenuates epileptic seizures in zebrafish and mouse models. Neurochemistry International. 2018; 112:124–33.
20. Daanaa S, Abotsi WKM, Boakye-Gyasi E, Woode E. Anticonvulsant effect of the hydroethanolic leaf extract of Psydrax subcordata (DC.) Bridson in murine models. Journal of Ethnopharmacology. 2018; 213:384–94.
21. De Caro C, Leo A, Nesci V, Ghelardini C, di Cesare Mannelli L, Striano P, et al. Intestinal inflammation increases convulsant activity and reduces antiepileptic drug efficacy in a mouse model of epilepsy. Sci Rep. 2019; 9(1):13983.
22. Dhamne SC, Silverman JL, Super CE, Lammers SHT, Hameed MQ, Modi ME, et al. Replicable in vivo physiological and behavioral phenotypes of the Shank3B null mutant mouse model of autism. Molecular Autism. 2017; 8(1):26.
23. Drexel M, Locker F, Kofler B, Sperk G. Effects of galanin receptor 2 and receptor 3 knockout in mouse models of acute seizures. Epilepsia. 2018; 59(11):e166–71.
24. Esmekaya MA, Tuysuz MZ, Tomruk A, Canseven AG, Yücel E, Aktuna Z, et al. Effects of cell phone radiation on lipid peroxidation, glutathione and nitric oxide levels in mouse brain during epileptic seizure. J Chem Neuroanat. 2016; 75(Pt B):111–5.
25. Fejgin K, Nielsen J, Birknow MR, Bastlund JF, Nielsen V, Lauridsen JB, et al. A Mouse Model that Recapitulates Cardinal Features of the 15q13.3 Microdeletion Syndrome Including Schizophrenia- and Epilepsy-Related Alterations. Biological Psychiatry. 2014; 76(2):128–37.
26. Fichna J, Socała K, Nieoczym D, Gach K, Perlikowska R, Janecka A, et al. The mu-opioid receptor-selective peptide antagonists, antanal-1 and antanal-2, produce anticonvulsant effects in mice. Progress in Neuro-Psychopharmacology and Biological Psychiatry. 2013; 40:126–31.
27. Gasior M, Yankura J, Hartman AL, French A, Rogawski MA. Anticonvulsant and proconvulsant actions of 2-deoxy-d-glucose. Epilepsia. 2010; 51(8):1385–94.
28. Gavzan H, Hashemi F, Babaei J, Sayyah M. A role for peroxisome proliferator-activated receptor α in anticonvulsant activity of docosahexaenoic acid against seizures induced by pentylenetetrazole. Neuroscience Letters. 2018; 681:83–6.
29. Guo L, Chen Y, Zhao R, Wang G, Friedman E, Zhang A, et al. Allosteric modulation of sigma-1 receptors elicits anti-seizure activities. British Journal of Pharmacology. 2015; 172(16):4052–65.
30. Hasebe N, Abe K, Sugiyama E, Hosoi R, Inoue O. Anticonvulsant effects of methyl ethyl ketone and diethyl ketone in several types of mouse seizure models. European Journal of Pharmacology. 2010; 642(1):66–71.
31. Hill-Yardin EL, Argyropoulos A, Hosie S, Rind G, Anderson P, Hannan AJ, et al. Reduced susceptibility to induced seizures in the Neuroligin-3R451C mouse model of autism. Neuroscience Letters. 2015; 589:57–61.
32. Hosseini-Zare MS, Salehi F, Seyedi SY, Azami K, Ghadiri T, Mobasseri M, et al. Effects of pentoxifylline and H-89 on epileptogenic activity of bucladesine in pentylenetetrazol-treated mice. European Journal of Pharmacology. 2011; 670(2):464–70.
33. Huang Q, Kuok KI, Zhang X, Yue L, Lee SMY, Zhang J, et al. Inhibition of drug-induced seizure development in both zebrafish and mouse models by a synthetic nanoreceptor. Nanoscale. 2018; 10(22):10333–6.
34. Huang W-Y, Lin S, Chen H-Y, Chen Y-P, Chen T-Y, Hsu K-S, et al. NADPH oxidases as potential pharmacological targets against increased seizure susceptibility after systemic inflammation. Journal of Neuroinflammation. 2018; 15(1):140.
35. Huang X, Zhou C, Tian M, Kang J-Q, Shen W, Verdier K, et al. Overexpressing wild-type γ2 subunits rescued the seizure phenotype in Gabrg2+/Q390X Dravet syndrome mice. Epilepsia. 2017; 58(8):1451–61.
36. Jayaraman R, Manisenthil KT, Anitha T, Joshi VD, Palei NN, Gajera K, et al. Influence of etoricoxib on anticonvulsant activity of phenytoin and diazepam in experimental seizure models in mice. J Pharm Pharmacol. 2010; 62(5):610–4.
37. Kopeikina E, Dukhinova M, Yung AWY, Veremeyko T, Kuznetsova IS, Lau TYB, et al. Platelets promote epileptic seizures by modulating brain serotonin level, enhancing neuronal electric activity, and contributing to neuroinflammation and oxidative stress. Prog Neurobiol. 2020; 188:101783.
38. Kyyriäinen J, Bolkvadze T, Koivisto H, Lipponen A, Pérez LO, Ekolle Ndode-Ekane X, et al. Deficiency of urokinase-type plasminogen activator and its receptor affects social behavior and increases seizure susceptibility. Epilepsy Research. 2019; 151:67–74.
39. Łączkowski KZ, Sałat K, Misiura K, Podkowa A, Malikowska N. Synthesis and anticonvulsant activities of novel 2-(cyclopentylmethylene)hydrazinyl-1,3-thiazoles in mouse models of seizures. Journal of Enzyme Inhibition and Medicinal Chemistry. 2016; 31(6):1576–82.
40. Lee S, Lee E, Kim R, Kim J, Lee S, Park H, et al. Shank2 Deletion in Parvalbumin Neurons Leads to Moderate Hyperactivity, Enhanced Self-Grooming and Suppressed Seizure Susceptibility in Mice. Front Mol Neurosci [Internet]. 2018 [cited 2020]; 11. Available from: https://www.frontiersin.org/articles/10.3389/fnmol.2018.00209/full
41. Leonetti A, Baroli G, Fratini E, Pietropaoli S, Marcoli M, Mariottini P, et al. Epileptic seizures and oxidative stress in a mouse model over-expressing spermine oxidase. Amino Acids. 2020; 52(2):129–39.
42. Li M, Milligan CJ, Wang H, Walker A, Churilov L, Lawrence AJ, et al. KCTD12 modulation of GABA(B) receptor function. Pharmacology Research & Perspectives. 2017; 5(4):e00319.
43. Lima RC de S, Silva MCC, Aguiar CCT, Chaves EMC, Dias KCF, Macêdo DS, et al. Anticonvulsant action of Calotropis procera latex proteins. Epilepsy & Behavior. 2012; 23(2):123–6.
44. Liu Z, Lindemeyer AK, Liang J, Wallner M, Shao XM, Shao Y, et al. Flavonoids isolated from Tibetan medicines, binding to GABAA receptor and the anticonvulsant activity. Phytomedicine. 2018; 50:1–7.
45. Loyens E, Vermoesen K, Schallier A, Michotte Y, Smolders I. Proconvulsive effects of oxytocin in the generalized pentylenetetrazol mouse model are mediated by vasopressin 1a receptors. Brain Research. 2012; 1436:43–50.
46. Luszczki JJ, Andres-Mach M, Barcicka-Klosowska B, Florek-Luszczki M, Haratym-Maj A, Czuczwar SJ. Effects of WIN 55,212-2 mesylate (a synthetic cannabinoid) on the protective action of clonazepam, ethosuximide, phenobarbital and valproate against pentylenetetrazole-induced clonic seizures in mice. Progress in Neuro-Psychopharmacology and Biological Psychiatry. 2011; 35(8):1870–6.
47. Ma J-Y, Quan Y-C, Jin H-G, Zhen X-H, Zhang X-W, Guan L-P. Practical Synthesis, Antidepressant, and Anticonvulsant Activity of 3-Phenyliminoindolin-2-one Derivatives. Chemical Biology & Drug Design. 2016; 87(3):342–51.
48. Mahendran G, Vijayan R. Neuropharmacological and molecular docking studies of xanthones from Swertia corymbosa. Journal of Receptors and Signal Transduction. 2018; 38(2):166–77.
49. Manville RW, Papanikolaou M, Abbott GW. M-Channel Activation Contributes to the Anticonvulsant Action of the Ketone Body β-Hydroxybutyrate. J Pharmacol Exp Ther. 2020; 372(2):148–56.
50. Menten-Dedoyart C, Navacerrada MES, Bartholome O, Gil JS, Neirinckx V, Wislet S, et al. Development and Validation of a New Mouse Model to Investigate the Role of SV2A in Epilepsy. PLOS ONE. 2016; 11(11):e0166525.
51. Mishra A, Punia JK, Bladen C, Zamponi GW, Goel RK. Anticonvulsant mechanisms of piperine, a piperidine alkaloid. Channels. 2015; 9(5):317–23.
52. Moezi L, Akbarian R, Niknahad H, Shafaroodi H. The interaction of adenosine and morphine on pentylenetetrazole-induced seizure threshold in mice. Neuropharmacology. 2013; 72:1–8.
53. Moezi L, Shafaroodi H, Hojati A, Dehpour AR. The interaction of melatonin and agmatine on pentylenetetrazole-induced seizure threshold in mice. Epilepsy & Behavior. 2011; 22(2):200–6.
54. Mukherjee S, Zeitouni S, Cavarsan CF, Shapiro LA. Increased Seizure Susceptibility in Mice 30 Days after Fluid Percussion Injury. Front Neurol [Internet]. 2013 [cited 2020]; 4. Available from: https://www.frontiersin.org/articles/10.3389/fneur.2013.00028/full
55. Ngoupaye GT, Ngo Bum E, Ngah E, Talla E, Moto FCO, Taiwe GS, et al. The anticonvulsant and sedative effects of Gladiolus dalenii extracts in mice. Epilepsy & Behavior. 2013; 28(3):450–6.
56. Nicolazzo JA, Steuten JA, Charman SA, Taylor N, Davies PJ, Petrou S. Brain uptake of diazepam and phenytoin in a genetic animal model of absence epilepsy. Clinical and Experimental Pharmacology and Physiology. 2010; 37(5–6):647–9.
57. Nieoczym D, Socała K, Gawel K, Esguerra CV, Wyska E, Wlaź P. Anticonvulsant Activity of Pterostilbene in Zebrafish and Mouse Acute Seizure Tests. Neurochem Res. 2019; 44(5):1043–55.
58. Orellana-Paucar AM, Afrikanova T, Thomas J, Aibuldinov YK, Dehaen W, Witte PAM de, et al. Insights from Zebrafish and Mouse Models on the Activity and Safety of Ar-Turmerone as a Potential Drug Candidate for the Treatment of Epilepsy. PLOS ONE. 2013; 8(12):e81634.
59. Orellana-Paucar AM, Serruys A-SK, Afrikanova T, Maes J, De Borggraeve W, Alen J, et al. Anticonvulsant activity of bisabolene sesquiterpenoids of Curcuma longa in zebrafish and mouse seizure models. Epilepsy & Behavior. 2012; 24(1):14–22.
60. Parker AK, Le MM, Smith TS, Hoang-Minh LB, Atkinson EW, Ugartemendia G, et al. Neonatal seizures induced by pentylenetetrazol or kainic acid disrupt primary cilia growth on developing mouse cortical neurons. Experimental Neurology. 2016; 282:119–27.
61. Payandemehr B, Bahremand A, Rahimian R, Ziai P, Amouzegar A, Sharifzadeh M, et al. 5-HT3 receptor mediates the dose-dependent effects of citalopram on pentylenetetrazole-induced clonic seizure in mice: Involvement of nitric oxide. Epilepsy Research. 2012; 101(3):217–27.
62. Pires LF, Costa LM, de Almeida AAC, Silva OA, Cerqueira GS, de Sousa DP, et al. Neuropharmacological effects of carvacryl acetate on δ-aminolevulinic dehydratase, Na+, K+-ATPase activities and amino acids levels in mice hippocampus after seizures. Chemico-Biological Interactions. 2015; 226:49–57.
63. Popławska M, Wróblewska D, Borowicz KK. Interactions between an antidepressant reboxetine and four classic antiepileptic drugs in the mouse model of myoclonic seizures. Pharmacol Rep. 2015; 67(6):1141–6.
64. Quraishi IH, Mercier MR, McClure H, Couture RL, Schwartz ML, Lukowski R, et al. Impaired motor skill learning and altered seizure susceptibility in mice with loss or gain of function of the Kcnt1 gene encoding Slack (K Na 1.1) Na + -activated K + channels. Scientific Reports. 2020; 10(1):3213.
65. Rahn JJ, Bestman JE, Josey BJ, Inks ES, Stackley KD, Rogers CE, et al. Novel Vitamin K analogs suppress seizures in zebrafish and mouse models of epilepsy. Neuroscience. 2014; 259:142–54.
66. Reyes-Marin KE, Nuñez A. Seizure susceptibility in the APP/PS1 mouse model of Alzheimer’s disease and relationship with amyloid β plaques. Brain Research. 2017; 1677:93–100.
67. Rigas P, Sigalas C, Nikita M, Kaplanian A, Armaos K, Leontiadis LJ, et al. Long-Term Effects of Early Life Seizures on Endogenous Local Network Activity of the Mouse Neocortex. Front Synaptic Neurosci [Internet]. 2018 [cited 2020]; 10. Available from: https://www.frontiersin.org/articles/10.3389/fnsyn.2018.00043/full
68. Ruskin DN, Svedova J, Cote JL, Sandau U, Rho JM, Jr MK, et al. Ketogenic Diet Improves Core Symptoms of Autism in BTBR Mice. PLOS ONE. 2013; 8(6):e65021.
69. Sałat K, Podkowa A, Kowalczyk P, Kulig K, Dziubina A, Filipek B, et al. Anticonvulsant active inhibitor of GABA transporter subtype 1, tiagabine, with activity in mouse models of anxiety, pain and depression. Pharmacological Reports. 2015; 67(3):465–72.
70. Sánchez-Elexpuru G, Serratosa JM, Sanz P, Sánchez MP. 4-PBA and metformin decrease sensitivity to PTZ-induced seizures in a malin knockout model of Lafora disease. Neuroreport. 2017; 28(5):268–71.
71. Sánchez‐Elexpuru G, Serratosa JM, Sánchez MP. Sodium selenate treatment improves symptoms and seizure susceptibility in a malin-deficient mouse model of Lafora disease. Epilepsia. 2017; 58(3):467–75.
72. Schallier A, Vermoesen K, Loyens E, Liefferinge JV, Michotte Y, Smolders I, et al. l-Theanine intake increases threshold for limbic seizures but decreases threshold for generalized seizures. Nutritional Neuroscience. 2013; 16(2):78–82.
73. Shaikh MohdF, Tan KN, Borges K. Anticonvulsant screening of luteolin in four mouse seizure models. Neuroscience Letters. 2013; 550:195–9.
74. Shekh‐Ahmad T, Mawasi H, McDonough JH, Finnell RH, Wlodarczyk BJ, Yavin E, et al. Enantioselective pharmacodynamic and pharmacokinetic analysis of two chiral CNS-active carbamate derivatives of valproic acid. Epilepsia. 2014; 55(12):1944–52.
75. Shmakova AA, Rubina KA, Rysenkova KD, Gruzdeva AM, Ivashkina OI, Anokhin KV, et al. Urokinase receptor and tissue plasminogen activator as immediate-early genes in pentylenetetrazole-induced seizures in the mouse brain. European Journal of Neuroscience. 2020; 51(7):1559–72.
76. Song M-X, Wang Z-Y, He S-H, Yu S-W, Chen S-L, Guo D-F, et al. Synthesis and Evaluation of the Anticonvulsant Activities of 4-(2-(Alkylthio)benzo[d]oxazol-5-yl)-2,4-dihydro-3H-1,2,4-triazol-3-ones. Molecules. 2018; 23(4):756.
77. Thomas NK, Willis S, Sweetman L, Borges K. Triheptanoin in acute mouse seizure models. Epilepsy Research. 2012; 99(3):312–7.
78. Todorov P, Rangelov M, Peneva P, Todorova N, Tchekalarova J. Anticonvulsant evaluation and docking analysis of VV-Hemorphin-5 analogues. Drug Development Research. 2019; 80(4):425–37.
79. Töllner K, Twele F, Löscher W. Evaluation of the pentylenetetrazole seizure threshold test in epileptic mice as surrogate model for drug testing against pharmacoresistant seizures. Epilepsy & Behavior. 2016; 57:95–104.
80. Tomaciello F, Leclercq K, Kaminski RM. Resveratrol lacks protective activity against acute seizures in mouse models. Neuroscience Letters. 2016; 632:199–203.
81. Tomonoh Y, Deshimaru M, Araki K, Miyazaki Y, Arasaki T, Tanaka Y, et al. The Kick-In System: A Novel Rapid Knock-In Strategy. PLOS ONE. 2014; 9(2):e88549.
82. Van Erum J, Valkenburg F, Van Dam D, De Deyn PP. Pentylenetetrazole-induced Seizure Susceptibility in the Tau58/4 Transgenic Mouse Model of Tauopathy. Neuroscience. 2020; 425:112–22.
83. Van Erum J, Van Dam D, De Deyn PP. PTZ-induced seizures in mice require a revised Racine scale. Epilepsy & Behavior. 2019; 95:51–5.
84. Vermoesen K, Serruys A-SK, Loyens E, Afrikanova T, Massie A, Schallier A, et al. Assessment of the convulsant liability of antidepressants using zebrafish and mouse seizure models. Epilepsy & Behavior. 2011; 22(3):450–60.
85. Warner TA, Smith NK, Kang J-Q. The therapeutic effect of stiripentol in Gabrg2+/Q390X mice associated with epileptic encephalopathy. Epilepsy Research. 2019; 154:8–12.
86. Willis S, Stoll J, Sweetman L, Borges K. Anticonvulsant effects of a triheptanoin diet in two mouse chronic seizure models. Neurobiology of Disease. 2010; 40(3):565–72.
87. Witsch J, Golkowski D, Hahn TTG, Petrou S, Spors H. Cortical alterations in a model for absence epilepsy and febrile seizures: In vivo findings in mice carrying a human GABA(A)R gamma2 subunit mutation. Neurobiology of Disease. 2015; 77:62–70.
88. 88. Wong JC, Dutton SBB, Collins SD, Schachter S, Escayg A. Huperzine A Provides Robust and Sustained Protection against Induced Seizures in Scn1a Mutant Mice. Front Pharmacol [Internet]. 2016 [cited 2020]; 7. Available from: https://www.frontiersin.org/articles/10.3389/fphar.2016.00357/full
89. Yamamura S, Hoshikawa M, Dai K, Saito H, Suzuki N, Niwa O, et al. ONO-2506 inhibits spike–wave discharges in a genetic animal model without affecting traditional convulsive tests via gliotransmission regulation. British Journal of Pharmacology. 2013; 168(5):1088–100.
90. Yang T, Kong B, Gu J-W, Kuang Y-Q, Cheng L, Yang W-T, et al. Anticonvulsant and sedative effects of paederosidic acid isolated from Paederia scandens (Lour.) Merrill. in mice and rats. Pharmacology Biochemistry and Behavior. 2013; 111:97–101.
91. Yuskaitis CJ, Jones BM, Wolfson RL, Super CE, Dhamne SC, Rotenberg A, et al. A mouse model of DEPDC5-related epilepsy: Neuronal loss of Depdc5 causes dysplastic and ectopic neurons, increased mTOR signaling, and seizure susceptibility. Neurobiology of Disease. 2018; 111:91–101.
92. Zangrandi L, Burtscher J, MacKay JP, Colmers WF, Schwarzer C. The G-protein biased partial κ opioid receptor agonist 6′-GNTI blocks hippocampal paroxysmal discharges without inducing aversion. British Journal of Pharmacology. 2016; 173(11):1756–67.
93. Zhang H, Zhao H, Feng H-J. Atomoxetine, a norepinephrine reuptake inhibitor, reduces seizure-induced respiratory arrest. Epilepsy & Behavior. 2017; 73:6–9.
94. 94. Zhang H, Zhao H, Yang X, Xue Q, Cotten JF, Feng H-J. 5-Hydroxytryptophan, a precursor for serotonin synthesis, reduces seizure-induced respiratory arrest. Epilepsia. 2016; 57(8):1228–35.
95. Zhang H, Zhao H, Zeng C, Van Dort C, Faingold CL, Taylor NE, et al. Optogenetic activation of 5-HT neurons in the dorsal raphe suppresses seizure-induced respiratory arrest and produces anticonvulsant effect in the DBA/1 mouse SUDEP model. Neurobiology of Disease. 2018; 110:47–58.
96. Zhao J, Tao H, Xian W, Cai Y, Cheng W, Yin M, et al. A Highly Selective Inhibitor of Glycine Transporter-1 Elevates the Threshold for Maximal Electroshock-Induced Tonic Seizure in Mice. Biological and Pharmaceutical Bulletin. 2016; 39(2):174–80.
97. Zolkowska D, Dhir A, Krishnan K, Covey DF, Rogawski MA. Anticonvulsant potencies of the enantiomers of the neurosteroids androsterone and etiocholanolone exceed those of the natural forms. Psychopharmacology. 2014; 231(17):3325–32.
